# Biodegradation of penicillin G from industrial bacteria residue by immobilized cells of *Paracoccus* sp. KDSPL-02 through continuous expanded bed adsorption bioreactor

**DOI:** 10.1186/s13036-020-0229-5

**Published:** 2020-02-22

**Authors:** Peng Wang, Chen Shen, Xiaochun Wang, Shouxin Liu, Luwei Li, Jinfeng Guo

**Affiliations:** 1 College of Chemical & Pharmaceutical Engineering, Hebei University of Science & Technology, Shijiazhuang, 050018 China; 2 State Key Laboratory Breeding Base-Hebei Province Key Laboratory of Molecular Chemistry for Drug, Hebei University of Science & Technology, Shijiazhuang, 050018 China; 3 Hebei Province Pharmaceutical Chemical Engineering Technology Research Center, Shijiazhuang, 050018 China

**Keywords:** Penicillin G, Expanded bed adsorption bioreactor, *Paracoccus* sp., Immolilization

## Abstract

**Background:**

An efficient biodegradation-strengthening approach was developed to improve penicillin G degradation from industrial bacterial residue in an expanded bed adsorption bioreactor (EBAB) is reported in this paper.

**Results:**

*Paracoccus* sp. strain KDSPL-02 was isolated based on its ability to use penicillin G as the sole carbon and nitrogen source. Strain identification was based on analyses of morphology, physio-biochemical characteristics, and 16S rDNA sequences. The effects of temperature, pH, PVA-sodium alginate concentration, calcium chloride concentration and initial penicillin G concentration were investigated. Repeated operations of immobilized cells with EBAB, At initial penicillin concentrations below 2.0 g L^− 1^, the continuous mode could reach more than 20 times, and the degradation rate reached 100%.

**Conclusions:**

The present study suggests that the EBAB system can be utilized for the simple and economical biodegradation of penicillin G from industrial bacterial residue.

## Introduction

In recent years, biomass resources, as a kind of renewable energy, have brought benefited to mankind. Among them, industrial bacterial residue from pharmaceutical industries has recently become widely used in some countries as fodder, which is rich in proteins and carbohydrates and used in animal husbandry [8]. However, new problems have arisen in the process of utilization. For instance, the typical antibiotic penicillin G from penicillin fermentation fungi residue (PFFR) may accumulate in the natural environments, in places such as soil, sediments, surface water, and groundwater, and may ultimately enter the food chain and the bodies of humans and animals; approximately 30–90% of unchanged penicillin G is excreted into waste systems [27]. Interestingly, at the trace residue concentrations in the sludge environment, antibiotics and their metabolites could also exhibit potential toxicity to humans and aquatic organisms and could be responsible for the emergence of antibiotic-resistant bacteria and genes. Thus, to reduce the risk of exposure, tools for effective degradation of penicillin residues in the environment are necessary [24].

PFFR is regarded as a hazardous solid waste that is disposed via approved methods, such ad landfills, incineration, anaerobic treatment, or composting. However, the waste mycelium contains large amounts of proteins, carbohydrates, and cellulose. Present strategies for the disposal of such wastes are costly and wasteful and cause serious secondary pollution [28]. Thorough studies have reported that microbes improve degradation in terrestrial and aquatic environments [12, 13]; however, the drawbacks of biodegradation include the formation of intermediates and incomplete mineralization. Several tentative suggestions have been proposed to solve this problem as follows. One solution involving heterogeneous photocatalysis is the coupling of advanced oxidation processes (AOPs) and biodegradation [1, 2, 15, 16]. Nevertheless, this is costly due to the multistep separation and is not easy to scale-up.

On the other hand, from an engineering standpoint, attempts were made in this study to treat real PFFR using biodegradation treatment followed by expanded bed adsorption bioreactor (EBAB). To the best of our knowledge this is the first reported study in which the EBAB system is being used in connection with the biodegradation process to treat real PFFR. An expanded bed adsorption bioreactor (EBAB) is products from in-situ separation processes of antibiotic degraded byproducts. Within this process, cell immobilization is a crucial step in degradation because of advantages such as improved tolerance to a variety of toxic and recalcitrant compounds in a suitable matrix [4, 18, 23, 25, 29], Immobilized bacterial cells are easy to separate and reuse and have higher volumetric reaction rates or higher local cell concentrations [3]. Therefore, immobilization could be the key in the biodegradation of industrial bacteria residues [5].

In the specific process of cell immobilization, cross-linked Ca-alginate is one of the most commonly used immobilization polymers due to its highly porous structure that facilitates the diffusion of solutes and dissolved gases [19]. However, alginate also has some problems in cell immobilization, such as low mechanical strength and restricted pH value application range. For these reasons, it is essential that research should focus on the investigation of an appropriate immobilized matrix that can be integrated into the cell immobilization process.

In the present study, we developed an economical and efficient strategy for penicillin G biodegradation. *Paracoccus* sp. strain KDSPL-02 was isolated from sludge contaminated by antibiotics based on its ability to use penicillin G as the sole carbon and nitrogen source. Strain identification was based on analyses of morphology, physio-biochemical characteristics, and 16S rDNA sequences. A polyvinyl alcohol (PVA)-alginate mixed matrix was used in the immobilization process for biodegradation. This study focused on determining the optimal immobilization conditions by single-factor tests. An expanded bed adsorption bioreactor (EBAB) was employed to degrade penicillin G residue. Repeated biodegradation properties in terms of the degradation rate and degradation time were also discussed.

## Materials and methods

### Chemicals

Sludge samples were collected from the district of Shijiazhuang, China. Penicillin G and PFFR were provided by North China Pharmaceutical Group Corporation, China. All other chemicals used in this study were of analytical grade and commercially available without further purification unless otherwise noted.

### Isolation and enrichment of penicillin G-degrading bacteria

The base mineral media (BMM) consisted of 1.60 g of K_2_HPO_4_, 0.40 g of KH_2_PO_4_, 0.20 g of MgSO_4_·7H_2_O, 0.03 g of CaCl_2_·2H_2_0, 0.02 g of FeCl_3_·6H_2_0, 0.50 g of NH_4_NO_3_, and 0.50 g of yeast extract per litre of water. The growth medium (GM) consisted of 2.40 g of yeast extract per litre of water. The final pH was adjusted to 7.0.

The bacterial strain was isolated from the collected sludge sample. Exactly 10 g (wet weight) of the sludge sample was added into 100 mL sterile BMM flasks, and 0.2 g L^− 1^ penicillin G was added as the inducer. The inoculated flasks were placed on an environmental orbital shaker at 120 rpm at 30 °C for 24 h. Then, 10 mL of the enrichment culture media mentioned above was transferred to 100 mL of fresh GM and cultivated under the same conditions. Afterwards, the enrichment suspension was streaked on nutrient agar plates and then incubated at 30 °C for 72 h. Colonies with a distinct morphological type were collected and transferred to standard agar medium until a pure strain was isolated. KDSPL-02 utilized penicillin G as the sole carbon and energy source for growth in BMM and thus was selected for further studies.

### Bacterial strain identification

KDSPL-02 was identified based on analyses of morphology, physio-biochemical characteristics, and 16S rDNA gene sequence. Physiological and biochemical identification of the strain was examined with reference to Bergey’s Manual of Determinative Bacteriology. Genomic DNA was prepared using standard procedures. Subsequently, 16S rDNA gene amplification, purification, and screening were performed as previously described [6]. The resulting sequence was compared with the GenBank nucleotide library via a BLAST search through the National Center for Biotechnology Information. Multiple alignments of 16S rDNA were carried out using CLUSTALX 1.8.1, and phylogeny was analyzed using MEGA4.0 [22]. A phylogenetic tree based on the 16S rRNA sequences was constructed using the neighbour-joining method after the alignment of related sequences from the GenBank database.

### Fermentation of free whole cell KDSPL-02

The inoculum was prepared by transferring a full loop of mycelia from a 2-day old suspension. The culture was incubated on a rotary shaker at 30 °C and 150 rpm for 48 h. For inoculation, 10% (v/v) spore suspension was transferred to 1 L Erlenmeyer flasks containing 200 mL of fresh fermentation medium and cultivated on an environmental orbital shaker at 120 rpm at 30 °C. After 48 h of incubation, the culture broth was centrifuged at 5000 rpm at 4 °C using a refrigerated centrifuge for further immobilization. The fermentation medium (FM) was composed of the following: 10 mL of industrial liquid sugar; 2.40 g of yeast extract, 1.60 g of K_2_HPO_4_, 0.40 g of KH_2_PO_4_, 0.20 g of MgSO_4_·7H_2_O, 0.03 g of CaCl_2_·2H_2_0, 0.02 g of FeCl_3_·6H_2_0, 0.50 g of NH_4_NO_3_ per litre of water.

### Immobilization of *Paracoccus* sp. KDSPL-02 cells

First, 2% sodium alginate in 100 ml of warm sterile distilled water (50 °C) was added to form a slurry completely, and the slurry was cooled to 30 °C. Second, 10 ml of cell suspension (OD_600_ = 1.2, wet weight 2.5 g) was placed in a sterile beaker, full of sodium alginate and PVA matrix slurry and subjected to a constant mild stirring. Third, the cell-alginate mixture was extruded dropwise through a needle (ID 2.0 mm) into 0.2 M CaCl_2_ solution containing sterile saturated boric acid solution by a peristaltic pump. Droplets were instantly transformed into spherical beads (2–3 mm in diameter). Finally, the beads were washed three times with sterile distilled water and stored in a refrigerator until further use.

### Optimal conditions of immobilization of *Paracoccus* sp. KDSPL-02 cells

For determining the optimal conditions for the immobilization of KDSPL-02 cells, a single-factor test was designed under different conditions of alternate temperature (15 °C–45 °C), pH (4.0–11.0), support materials and cross-linking solution concentration, initial tolerance concentration of penicillin G (1.2 and 1.6 g L − 1). KDSPL-02 was incubated in GM at 120 rpm and 30 °C on an orbital shaker. Each treatment was set in triplicate with free cell samples as control.

### EBAB construction and penicillin G degradation process

**Fig. 1:**
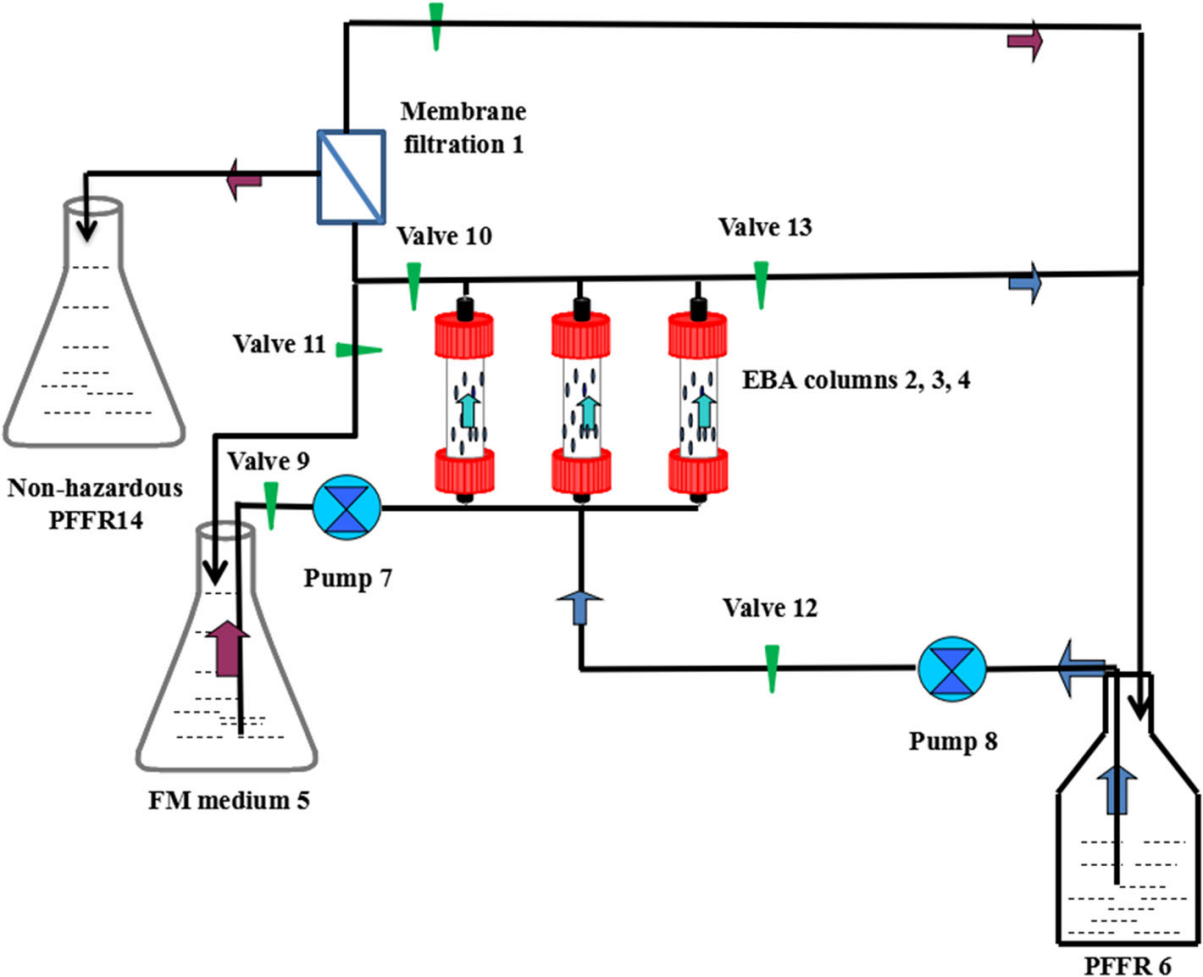
Schematic dragram of the EBAB system used in penicillin G biodegradation

The EBA-based bioreactor was mainly an external recovery loop composed primarily of a roll membrane filtration (relative molecular mass 200; purchased from Jinhua Co., Shanghai, China) (Fig. 1, detail 1) and three parallel EBA columns (25 mm o.d., length 500 mm, total volume 200 mL; purchased from Jinhua Co., Shanghai, China) (Fig. 1, detail 2, 3 and 4). The external recovery loop was established by connecting the EBA columns to the storage vat (Fig. 1, detail 5 and 6) with Teflon tubing (4.5 mm o.d., 3.0 mm i.d.; Hamilton, Bonaduz, Switzerland). Pharmed BPT tube (2.5 mm o.d., 2.4 mm i.d.; Cole-Parmer, USA) was used to integrate the peristaltic pump (Cole-Parmer, USA) (Fig. 1, detail 7 and 8) into the loop. To retain the immobilized particles within the column while allowing the passage of the cells, both ends were equipped with a wire mesh (stainless steel; diameter 20 mm, 250 μm mesh width) fixed with a Teflon ring. The openings of the column connectors had an inner diameter of 4 mm.

Repeated degradation was performed in the EBAB system on the basis of the optimal immobilization approach. The operation consisted of three steps. First, valves 9, 10 and 11 were opened and the immobilized cells were precultured in an expanded bed column with fresh medium. Second, valve 9, 10 and 11 were closed, pump 8 was started, and values 12 and 13 were opened. The PFFR suspension was circulated through the EBA column for biocatalytic degradation in the expanded bed. For operation of the expanded bed, the PFFR suspension flowed through the bedpost at a rate of 4 L h^− 1^. When the catalytic activity of the immobilized particle decreased, columns were alternated and switched on to their corresponding spigots were switched on to repeatedly operate the circulation system. The PFFR suspension through the cylinder circulated back to storage vat 14. When the residual antibiotic concentration in PFFR fell below the nonhazardous concentration, the third step was started, valves 10, 12 and 14 were opened, valve 13 was closed and pump 8 was started. The nonhazardous PFFR suspension was filtered through membrane filtration, the filtrate was recycled, and the concentrated nonhazardous PFFR suspension entered storage vat 14.

Through this process, the immobilized cells were activated by a moderate amount of fresh FM, and then the industrial bacterial residue was dissolved in water containing penicillin G and passed through membrane filtration. The clear liquid was circulated through the EBA column with immobilized cells, and penicillin G was degraded in the expanded bed column by the continuous mode at specific time intervals. The expanded bed was operated such that the liquid flowed at a rate of 150 mL/h. The columns were alternated and switched to their corresponding valves to repeatedly operate the circulation system after biocatalytic degradation.

### Analytical methods

Concentration of penicillin G was determined by using high performance liquid chromatography (HPLC) system, with an Ultimate XB C18 column (4.6 × 250 mm, 5 μm). The detailed chromatography conditions were carried out as following. The samples were measured with a flow rate of 1.0 mL/min; Column temperature: 30 °C, mobile phase: methanol/phosphate (0.1 M potassium dihydrogen phosphate, pH, flow rate: 1.0 mL/min, injection volume: 20 μL; detection wavelength: 225 nm.

### Penicillin G degradation conditions

#### Degradation by free cells

A 0.2 mL suspension of whole cell KDSPL-02 with a degradation activity of 2–7.0 U was added to a 250 mL conical flask containing a 100 mL solution of 0.8–1.6 g L^− 1^ penicillin G. The activity (U) of penicillin G degraded by KDSPL-02 free cells were defined as: g g^− 1^ h^− 1^, the wet cell of one gram degraded penicillin (g) at 1 h.

#### Degradation by immobilized cells

Polymer beads (without cells) inoculated to the sterile distilled water served as control to investigate the removal of Penicillin G by adsorption to the immobilized beads. The activity (U) of penicillin G degraded by KDSPL-02 immobilized cells was defined as: g g^− 1^ h^− 1^, the immobilized wet cell of one gram degraded penicillin (g) at 1 hour.

## Results and discussion

### Isolation and characterization of penicillin G-degrading strains

Using penicillin G as the sole carbon source, bacteria that can degrade penicillin G were successfully enriched. After 2 weeks of acclimatization, six single colonies with different morphological types were isolated and identified as KDSPL-01–KDSPL-06. A pilot study showed that KDSPL-02, without any treatment, exhibited the highest penicillin G degradation ability. Therefore, it was chosen as the target strain. The purity of KDSPL-02 was determined by the morphological uniformity of cells from a single colony on solid medium plates via microscopic observation.

The morphology of KDSPL-02 cells was observed under a microscope. The colonies of KDSPL-02 appeared circular and light yellow with regular margins during growth on GM plates for 48 h. KDSPL-02 cells were spherical and free of flagella. Colonies were round, pale-yellow, and glossy with a diameter of 0.5–0.9 μm on agar plates. Based on physiological and biochemical tests, the strain was positive in physiological and biochemical tests for adonitol, L-arabinol, ELLMAN reagents, tyrosine aramidase, and the utilization of D-tagatose. The strain was negative for Gram staining, alanine phenylalanine, D-cellobiose, glutamine synthase, β-glucosidase, and the utilization of D-glucose and β-alanine aminolase (detailed physiological and biochemical characteristics are presented in Table 1).

**Table 1:** Morphological and biochemical characteristics of KDSPL-02

Characteristics	Results	Characteristics	Results
O-Xylosidase	–	D-cellobiose	–
Ornithine decarboxylase	–	Lipase	–
Citrate	–	O-Alanine aminolase	–
O-Glucosidase	–	Tyrosine aramidase	+
D-glucose	–	L-pyrrolidinyl arylamine	–
Malonate	–	H2S production	–
Adonitol	+	γ-glutamyltransferase	–
Phosphatase	–	sucrose	–
L-Arabinol	+	Glutamine synthase	–
D-tagatose	+	β-glucosidase	–
D-trehalose	–	β-Galactodase	–
ELLMAN reagent	+	Lysine decarboxylase	–
D-maltose	+	O-Galactosidase	–

**Fig. 2:**
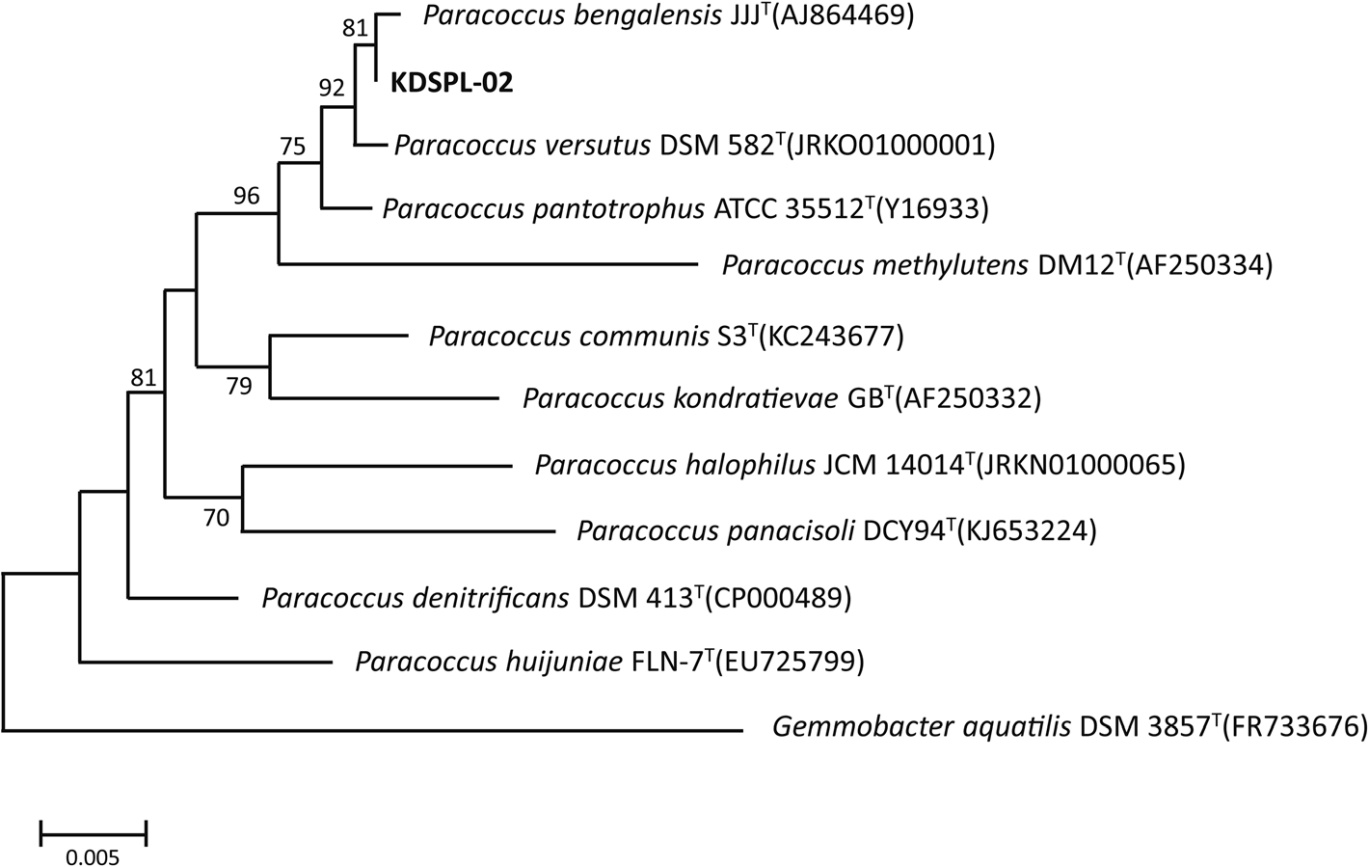
Phylogenetic analysis of the strain KDSPL-02 related species by the neighbour-joining approach. The scale bars represent 0.005 substitutions per site

The 16S rDNA gene from KDSPL-02 was PCR amplified, and a single fragment of 1315 bp, was obtained and completely sequenced. According to BLAST analysis, the resulting sequence had high similarity to the 16S rDNA gene sequence of bacteria belonging to the *Paracoccus* group and closely clustered with strain DSM582^T^ (GenBank accession no. JRKO 01000001) and strain JJJ (GenBank accession no. AJ 864469), with sequence identities of 99.92 and 99.92%, respectively. A phylogenetic tree was constructed based on the 16S rDNA gene sequence of KDSPL-02 and related strains using MEGA 4.0 (Fig. 2). In consideration of the morphological, physio-biochemical, and 16S rDNA gene analyses, KDSPL-02 was tentatively identified as *Paracoccus* sp*.*

Bacteria such as *Pseudomonas* and *Escherichia coli* are metabolically active microorganisms capable of degrading many antibiotics isolated from various soils or sediments [9, 11]. Meanwhile, *Paracoccus* sp*.* is a new bacterial genus that may participate in the efficient degradation of penicillin G. To the best of our knowledge, there is no information concerning the ability of *Paracoccus* sp. to degrade penicillin G*.* Furthermore, the potential enzymes such as beta-lactamases and penicillin acylases were predicted by searching NCBI database and There are 892 beta-lactamase and 156 penicillin acylases from *Paracoccus* sp. when searching from NCBI database. Several beta-lactamases and penicillin acylases from *Paracoccus* sp. were listed in Table S1 and Table S2, respectively.

### Optimization of immobilized of KDSPL-02 cells

#### Effect of temperature on immobilized KDSPL-02 cells

**Fig. 3:**
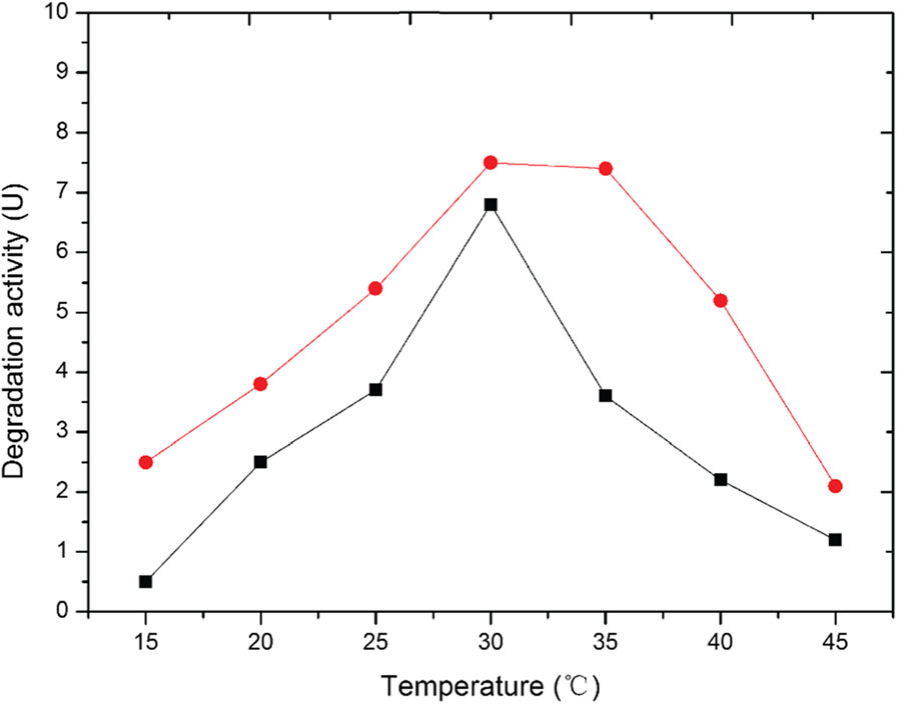
The influence of temperature on the degradation activity of free and immobized KDSPL-02 cell. (black square refers free cells, red circle refers immobilized cells)

The effect of temperature on the degradation activity of free and immobilized *Paracoccus* sp. KDSPL-02 cells was investigated at various temperatures (15 °C–45 °C) (Fig. 3). The optimum temperatures studied for the degradation activity by *Paracoccus* sp. KDSPL-02 were determined. The results showed that free and immobilized cells exhibited optimum degradation activity at different temperatures. The optimum temperature of the free cells was 30 °C, while the optimum temperature of the immobilized cells ranged from 30 °C–35 °C, which implied that immobilized cells could function in a broader temperature environment. According to the above result, the experimental temperature was 30 °C in the following test.

#### Effect of pH on immobilized KDSPL-02 cells

**Fig. 4:**
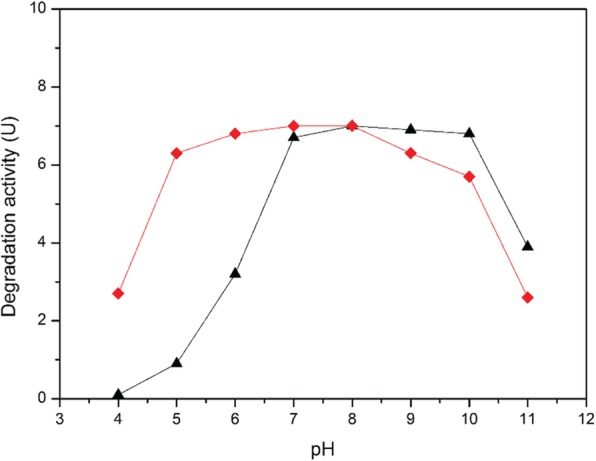
Effect of pH on the degradation ability of the immobilized KDSPL-02. (black triangle refers free cells, red circle refers immobilized cells)

The pH of the medium is an important factor that can affect cell growth since it influences the metabolic pathways, the activity of degradation and the dominating species in a mixed culture [10]. Variations in the pH of the fermentation medium can affect the penicillin G-degrading microbial population in a mixed microflora and even the cellular structure and morphology of the microbes [21]. However, due to support materials also enhancing the stability of the immobilized cell, the immobilization process successfully in improved the cell pH tolerance. We compared the stability and tolerance of free and immobilized cells at various pH values. The analysis carried out in this study demonstrates substantial differences in the pH optima of free and immobilized cells. The data clearly depicted that free cells can normally grow from pH 6 to 10, and the optimum pH was 7 to 8; the cells started losing its activity at pH 8, but immobilization of cells in alginate beads expanded the range of normal growth from pH 5 to 10, and activity was retained up to pH 9 (Fig. 4). The shifting of the pH towards an alkaline value upon immobilization may be due to secondary interactions between the enzyme and the polymer matrix. In addition, the polar groups of alginate may have interacted with the functional groups of the cells, which changed the pH characteristics of the enzyme. These cases also appear in the other support materials.

#### Effect of support materials and cross-linking solution concentration on immobilized KDSPL-02 cell cells

**Fig. 5:**
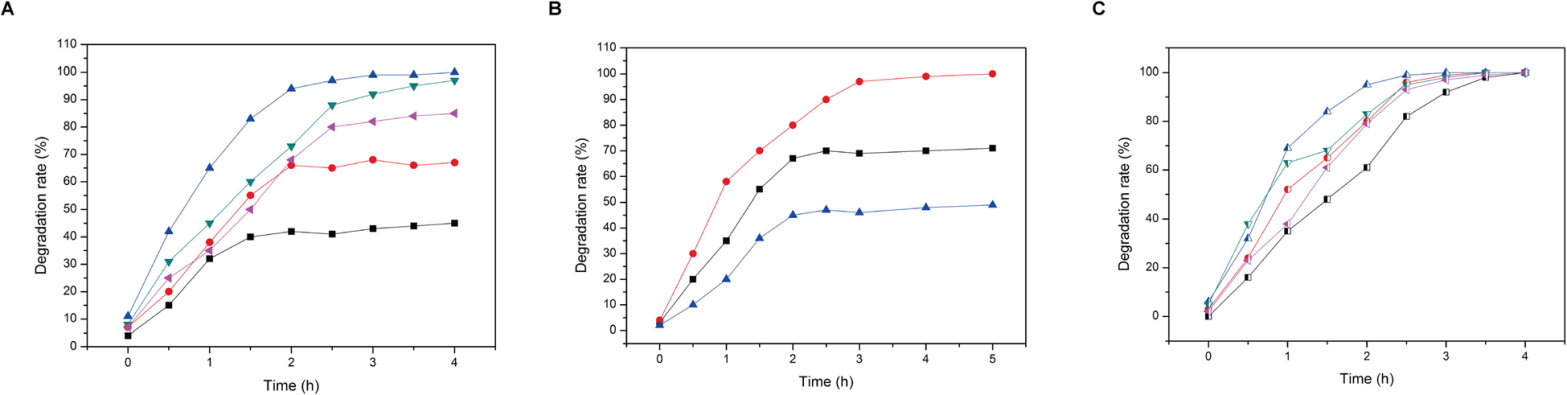
Effect of support materials and cross-linking solution concentration on the formation of immobilized beads. **a** Effect of PVA concentration on the formation of immobilized beads (black square refers 2.0% PVA, red circle refers 2.5% PVA, blue up triangle refers 3.0% PVA, green down triangle refers 3.5% PVA, pink left triangle refers 4.0% PVA); **b** Effect of calcium chloride concentration on the formation of immobilized beads (black square refers 1.8% calcium chloride, red circle refers 2.0% calcium chloride, blue triangle refers 2.2% calcium chloride); **c** Effect of sodium alginate concentration on the formation of immobilized beads (black square refers 2.0% sodium alginate, red circle refers 2.5% sodium alginate, blue up triangle refers 3.0% sodium alginate, green down triangle refers 3.5% sodium alginate, pink left triangle refers 4.0% sodium alginate)

To developing stable beads to impart better entrapment efficiency of cells, removal ability of the immobilized KDSPL-02 cells and improved penicillin G tolerance of cells, different concentrations of support materials and cross-linking solution were evaluated. The degradation activity decreased when the sodium alginate concentration was below 3%(w/v) due to the large pore size causing leakage of cells. The immobilized beads were transparent and soft (Fig. 5a). The degradation activity decreased at concentrations higher than 4% (w/v) as the pore size decreased, resulting in hindrance of substrate penetration into beads, and immobilized balls were opaque, hard and trailing. As reported previously, at sodium alginate concentration of 4%, immobilized beads that cause steric hindrance that intensified the interaction between the active site of the cell and alginate were produced, thus possibly causing higher levels of binding between the cell and sodium alginate, which resulted in poor degradation [26]. We finally chose a concentration of 3%(w/v), and proceeded with this concentration in further experiments. An observation of the PVA-alginate blended matrix was made (Fig. 5b). The concentration of the cross-linking solution was directly related to the bead intensity and stability. When the concentration of cross-linking solution was low, the immobilized beads were soft and easily broken; when the cross-linking solution concentration was higher, the microbial cell activity was reduced due to the high osmotic pressure of salt, which caused cell dehydration and reduction in microbial activity. The results showed that 2% of calcium chloride concentration solution performed well for cross linkage (Fig. 5c). Recently, various support materials have been used for cell immobilization such as metal organic framework [14], zeolite [7], iron oxide nanoparticles [17, 20] and so on. In our further studies, more support materials and cross-linking solution will be studied for enhancing the biodegradation efficiency.

#### Effect of the initial concentration of penicillin G on immobilized KDSPL-02 cells

**Fig. 6:**
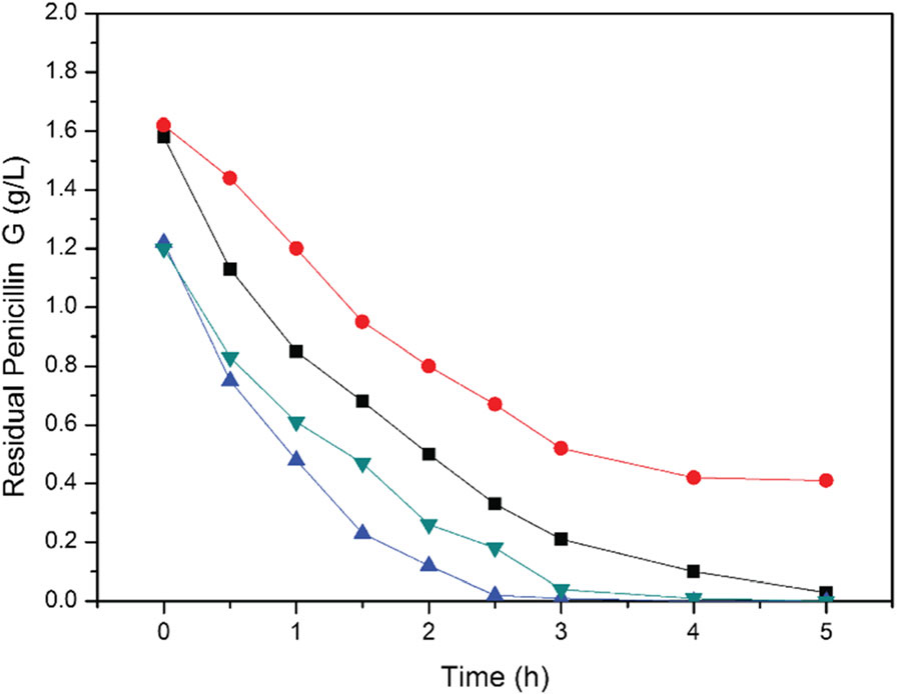
Degradation of free and immobilized cells with different initial concentrations of Penicillin G. (black square refers 1.6 g/L immobilized cells, red circle refers 1.6 g/L free cells, blue up triangle refers 1.2 g/L immobilized cells, green down triangle refers 1.2 g/L free cells)

To determine the tolerance limits of free and immobilized cells, different concentrations (1.2 g L^− 1^ and 1.6 g L^− 1^) of penicillin G were applied (Fig. 6). The experimental results showed that when the initial concentration of penicillin was 1.2 g L^− 1^, free and immobilized KDSPL-02 cells could degrade penicillin in 5 h only the degradation activity of immobilized cells was higher than that of free cells. However, when the initial concentration of penicillin was 1.6 g L^− 1^, not only was the degradation activity of immobilized cells was higher than that of free cells, but also the degradation activity of immobilized cells was higher than that of free cells. After 5 h of degradation, the degradation process of free cells tended to stop. As expected, immobilized cells contributed to gaining the properties of support materials. The support materials not only contributed to the stability and mechanical strength of the beads but also contributed to the improvement of degradation efficiency and tolerance limits.

The application of the biodegradation has been hampered due to low stability at higher temperature and extreme pH conditions, difficulty in recovery and reusability. After immobilization, the operational stability (such as thermal, pH stability, chemical stability as well as their storage stability) of enzymes profoundly increases which helps to extend their applications.

### Continuous degradation of penicillin G with the EBAB system

**Fig. 7:**
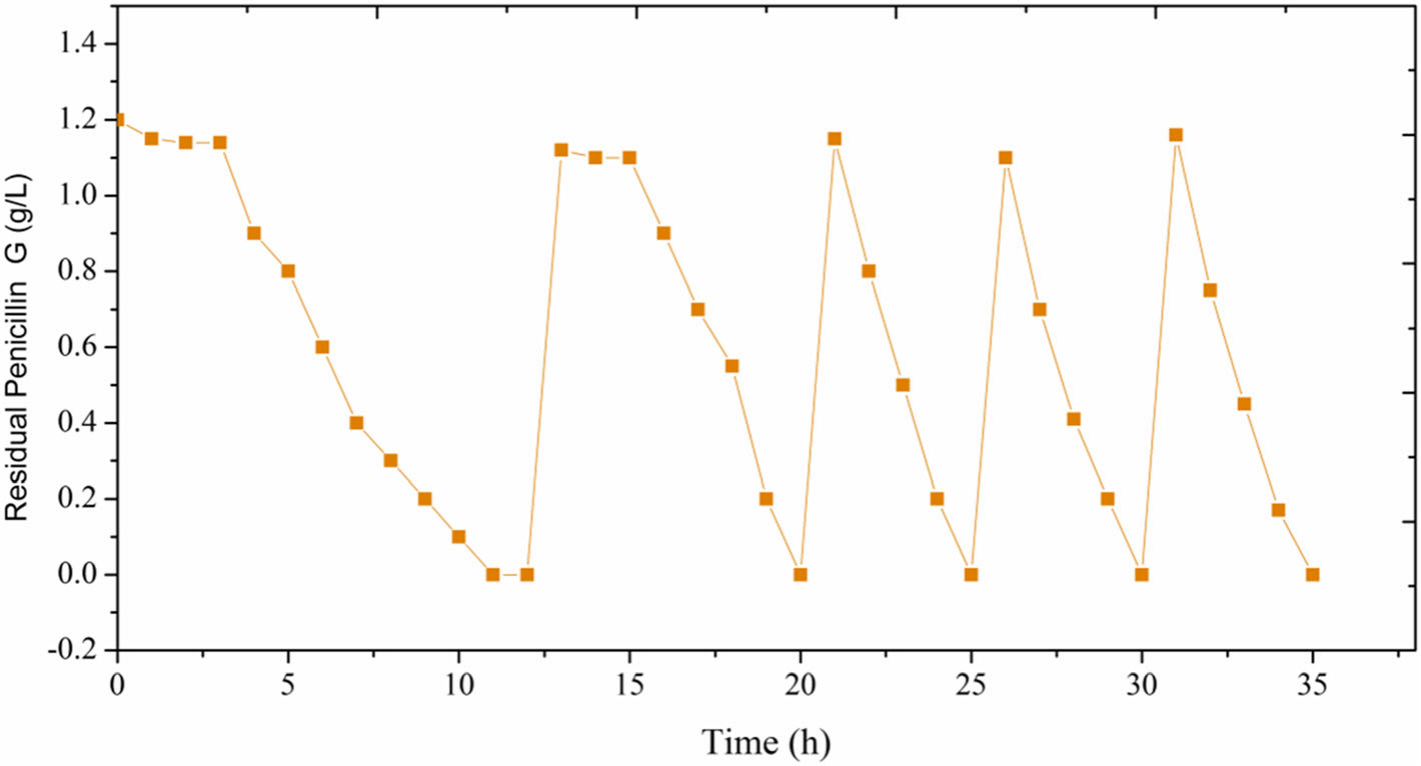
Repeated batch Penicillin G biodegradation in EBAB system. Biodegradation conditions: 30 °C, pH 7.0, 3.0% sodium alginate, 3.0% PVA and 2.0% calcium chloride. Initial Penicillin G concentration was 1.2 g L^−1^

Based on the above results, we employed the optimal strategy foe immobilization of KDSPL-02 cells for repeated penicillin G biodegradation by the EBAB system. The continuous degradation studies were carried out in a batch bioreactor containing 2.0 kg of beads (100 g of beads immobilized wet weight of 2.0 g, OD _600_ = 3.0 cell suspension). The kinetic profile of the repeated batch degradation is shown in Fig. 7. In the first batch, a 3 h lag phase was observed, and then the penicillin G concentration decreased rapidly. After 12 h of degradation, penicillin G was completely degraded. Repeated batch cultures were performed using the method described in Section 2.7. The lag phase was eliminated in batch 2 because the cells from batch 1 were reused to inoculate the next batch. Penicillin G was removed immediately, and the degradation time shortened from 12 h (batch 1) to 8 h (batch 2). No lag phase was observed in batch 3. The fermentation time was only 5 h. The degradation efficiency was maintained constant in the following repeated culture cycles of batches 4 and 5. As described in Table 2, the fermentation time significantly shortened from 12 h (for batch 1) to 5 h (for batch 5).

**Table 2:** Repeated operations of free and immobilized cells on penicillin G biodegradation

Matrix	Initial penicillin G (g L^−1^)	Degradation rate (%)	Degradation time (h)	No. of cycles
Immobilized	1.0	100	4	24
	1.5	100	5	24
	2.0	100	6	20
	2.5	90	10	20
	3.0	79	20	18
Free	1.0	100	12	10
	1.5	100	15	10
	2.0	95	20	8
	2.5	88	24	6
	3.0	80	30	6

**Fig. 8:**
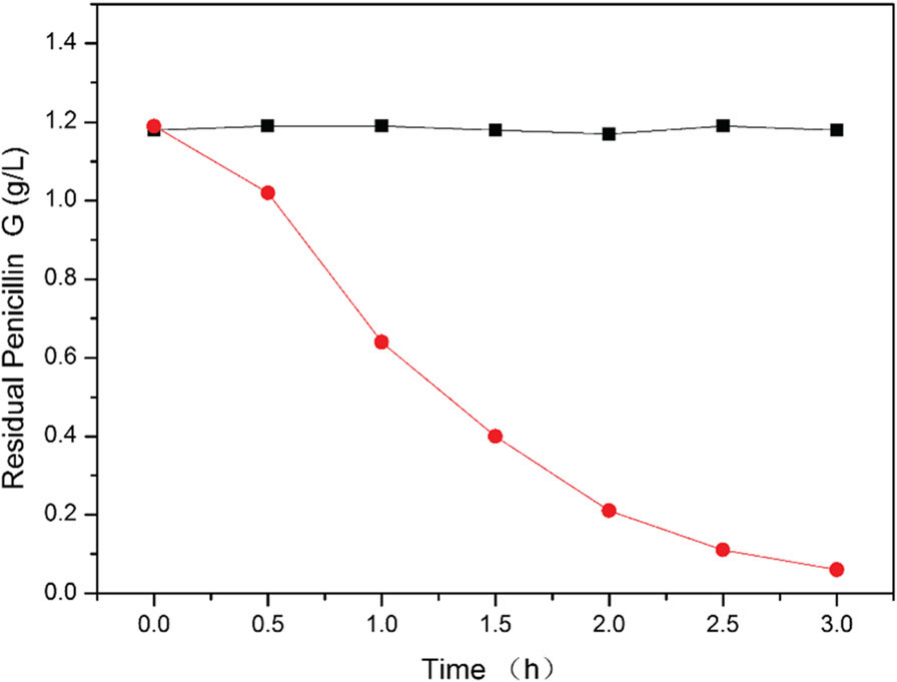
Penicillin G degradation activity of clear liquid and crude liquid in expanded bed reactor. (black square refers clear liquid, red circle refers crude liquid)

To prevent the loss of degrading bacteria, we recycled the bacterial suspension in the EBAB. After filtering with an ultrafiltration membrane, we tested the degradation activity of crude liquid and clear liquid (Fig. 8) and found that the clear liquid had no degradation activity for penicillin, which proves that we can effectively prevent the loss of degrading bacteria, and further implies that this EBAB-based biocatalytic process could avoid a series of biological safety problems caused by the loss of antibiotic degradation plasmids.

### Repeated operations of immobilized cells

One of the most important advantages of immobilized cell systems is the extended and repeated use of the biocatalyst for sequential batches. Experiments conducted for repeated operations of immobilized cells with EBAB are shown in Table 2. The experimental results showed that when the initial concentration of penicillin was below 2.0 g L^− 1^, the process could be repeated more than 20 times, and the degradation rate reached 100%. When the initial concentration of penicillin was above 2.0 g L^− 1^, the degradation rate decreased and the batch degradation time was correspondingly prolonged. In addition, using free-cell mode, the number of repeated operations decreased and the degradation time was prolonged under the same initial penicillin concentration.

## Conclusions

In summary, the novel bacterial strain *Paracoccus* sp. KDSPL-02 was isolated and identified. This strain was identified based on analyses of morphology, physio-biochemical characteristics, and 16S rDNA gene sequences. Further, *Paracoccus* sp. KDSPL-02 cells entrapped in sodium alginate beads, PVA-alginate beads showed more tolerance to penicillin G. These entrapped cells have effectively removed penicillin G from the industrial bacterial residues. These biocatalysts were used both in semi continuous and continuous mode to remove penicillin G from effluents collected from pharmaceutical industries. When the initial concentration of penicillin was below 2.0 g L^− 1^, the process could be repeated more than 20 times, and the degradation rate reached 100%. The present study clearly demonstrates the development of an industrially feasible and economically viable bioremediation strategy for discharging penicillin G-free effluents into the environment.

## Supplementary information

**Additional file 1: Table S1.** beta-lactamases in Paracoccus sp. Species. **Table S2.** penicillin acylases in Paracoccus sp. Species.

## Data Availability

The datasets used and/or analysed during the current study are available from the corresponding author on reasonable request.

## Abbreviations

BLAST: Basic Local Alignment Search Tool
EBAB: Expanded bed adsorption bioreactor
PFFR: Penicillin fermentation fungi residue
