# Supplementary material for: Biodegradation of penicillin G from industrial bacteria residue by immobilized cells of Paracoccus sp. KDSPL-02 through continuous expanded bed adsorption bioreactor
Source: J Biol Eng. 2020 Feb 22;14:5. doi: 10.1186/s13036-020-0229-5 (PMC7036172; doi:10.1186/s13036-020-0229-5)
Supplement: Supplementary file 1 — Additional file 1: Table S1. beta-lactamases in Paracoccus sp. Species. Table S2. penicillin acylases in Paracoccus sp. Species. [file 13036_2020_229_MOESM1_ESM.docx]

**Supplementary materials**

**Biodegradation of Penicillin G from industrial bacteria residue by immobilized cells of *Paracoccus* sp. KDSPL-02 through continuous expanded bed adsorption bioreactor**

Peng Wang^1,2,3*^ Chen Shen^2^ Xiaochun Wang^1^  Shouxin Liu^1,2^* Luwei Li^1^ and Jinfeng Guo^1^

^1^ College of Chemical & Pharmaceutical Engineering, Hebei University of Science & Technology, Shijiazhuang, 050018, China

^2^ State Key Laboratory Breeding Base-Hebei Province Key Laboratory of Molecular Chemistry for Drug, Hebei University of Science & Technology, Shijiazhuang, 050018, China

^3^ Hebei Province Pharmaceutical Chemical Engineering Technology Research Center, Shijiazhuang, 050018, China

* Correspondence: [pwang@hebust.edu.cn](mailto:pwang@hebust.edu.cn), [hebust_lsx@126.com](mailto:hebust_lsx@126.com)

Table S1 beta-lactamases in *Paracoccus sp.* Species

| No. of aa. | sources | Accession number |
| --- | --- | --- |
| 378 | *Paracoccus aminovorans* | CQR87441.1 |
| 377 | *Paracoccus denitrificans* PD1222 | ABL70748.1 |
| 377 | *Paracoccus versutus* | KGJ11682.1 |
| 427 | *Paracoccus aminophilus* JCM 7686 | AGT08961.1 |
| 297 | Paracoccus sanguinis | KGJ23694.1 |

Table S2 penicillin acylases in *Paracoccus sp.* Species

| No. of aa. | sources | Accession number |
| --- | --- | --- |
| 818 | *Paracoccus denitrificans* | GEK67130.1 |
| 919 | *Paracoccus sanguinis* | KGJ18942.1 |
| 817 | *Paracoccus halophilus* | KGJ06198.1 |
| 818 | *Paracoccus versutus* | KGJ10535.1 |
| 805 | *Paracoccus sp*. 228 | KIX18685.1 |
